# Supplementary material for: APOGEE 2: multi-layer machine-learning model for the interpretable prediction of mitochondrial missense variants
Source: Nat Commun. 2023 Aug 19;14:5058. doi: 10.1038/s41467-023-40797-7 (PMC10439926; doi:10.1038/s41467-023-40797-7)
Supplement: Supplementary file 1 — Supplementary Information [file 41467_2023_40797_MOESM1_ESM.pdf]

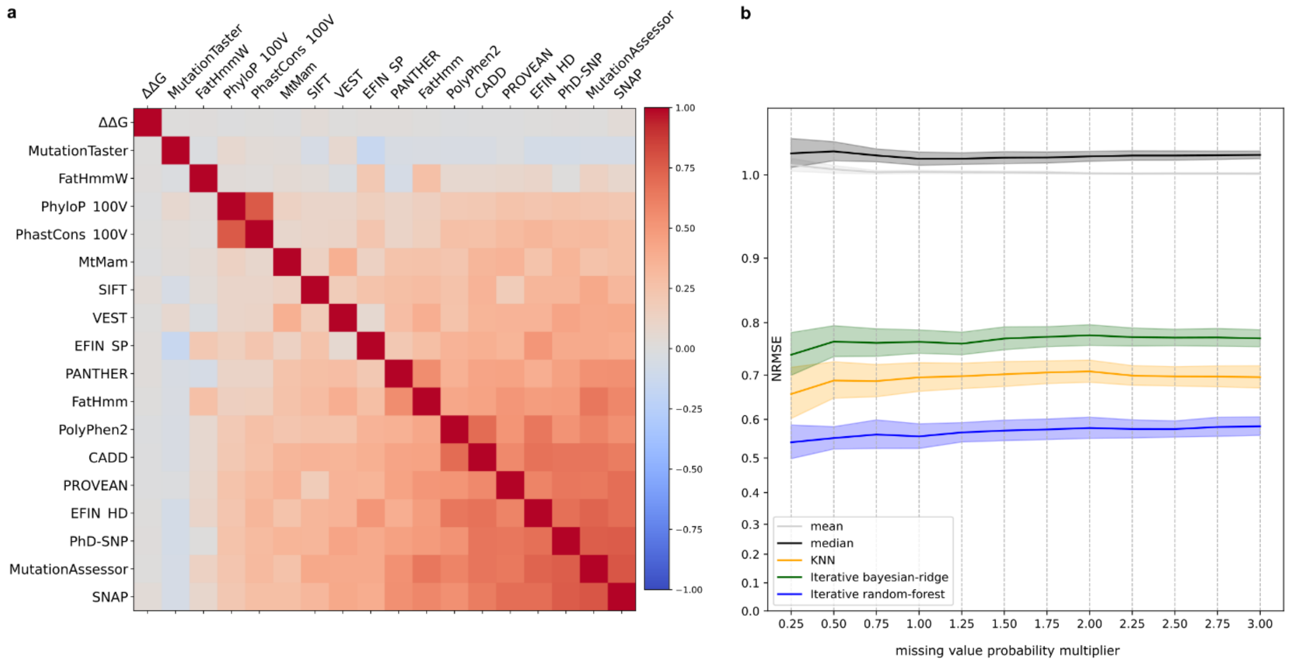

Supplementary Figure 1. Pairwise feature correlation and imputation of missing data. **a.** Matrix of Pearson correlation coefficients calculated on the scaled prediction scores to zero mean and unit variance of non-positional features for all possible mitochondrial missense variants. Axes labels related to pathogenicity predictors are highlighted in bold. **b.** Imputation of missing values with five algorithmic strategies; performance is measured in terms of Normalized Root Mean Squared Error (NRMSE, Y-axis); multipliers of the *a priori* probability of missing values in MitImpact (i.e., the unitary value) on the X axis (see Methods); both mean errors (lines) and their 95% CI (colored shadows) are reported in the figure.

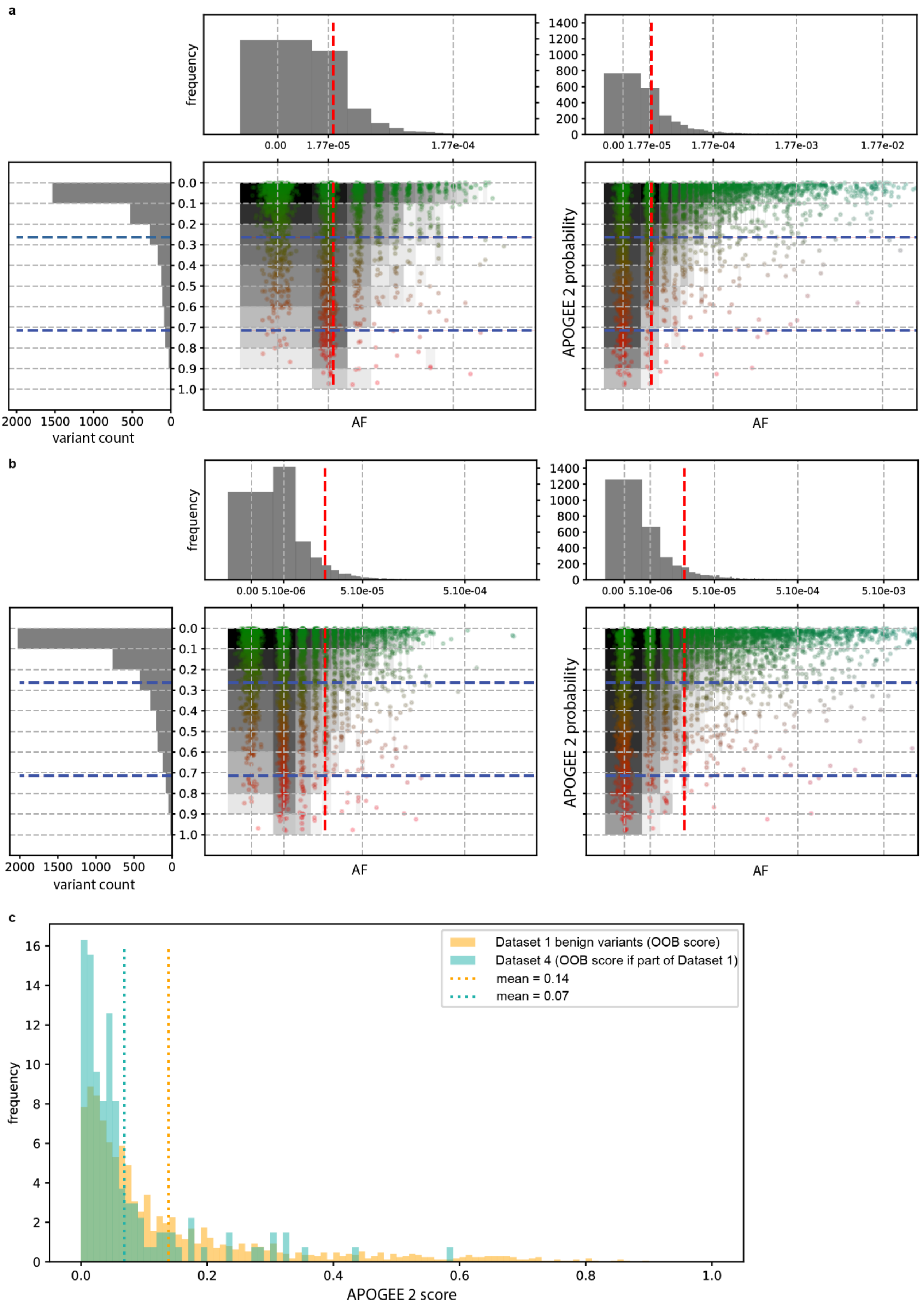
a
frequency
1400
1200
1000
800
600
400
200
0
0.00
1.77e-05
1.77e-04
1.77e-03
1.77e-02
0.0
0.1
0.2
0.3
0.4
0.5
0.6
0.7
0.8
0.9
1.0
2000 1500 1000 500 0
variant count
AF
APOGEE 2 probability
b
5.10e-06
5.10e-05
5.10e-04
5.10e-03
c
Dataset 1 benign variants (OOB score)
Dataset 4 (OOB score if part of Dataset 1)
mean = 0.14
mean = 0.07
16
14
12
10
8
6
4
2
0.2
0.4
0.6
0.8
1.0
APOGEE 2 score

Supplementary Figure 2. Distribution of heteroplasmic and homoplasmic variants in population and clinical databases. **a.** 83 (4.7%) heteroplasmic variants in gnomAD were predicted to be likely pathogenic, and 10 (0.6%) were pathogenic. 71 (4%) and 7 (0.4%) variants exhibited an AF <0.002%, respectively. 19 (0.9%) homoplasmic variants were classified as likely pathogenic and 3 (0.1%) as pathogenic. 11 (0.5%) and 1 (0.05%) variants exhibited an AF <0.002 %, respectively. **b.** 101 (3.3%) HelixMTdb heteroplasmic variants were predicted as likely pathogenic, and 15 (0.5%) were classified as pathogenic. Similarly, 33 (1.1%) homoplasmic variants were defined as likely pathogenic, and 3 (0.1%) were deemed pathogenic. 86 (2.8%) heteroplasmic and 22 (0.8%) homoplasmic likely pathogenic variants exhibited an AF <0.002%; 13 (0.4%) and 2 (0.07%) considering heteroplasmic and homoplasmic pathogenic variants, respectively. Both panels represent the APOGEE 2 pathogenicity probability values of heteroplasmic (left) and homoplasmic (right) variants on the Y axis and the variant AF on the X axis. A dot is a variant. A dot color represents the pathogenicity level (green = benign, red = pathogenic), as in Figure 3D. The red dashed line marks the 0.002% AF threshold. The blue dashed lines separate likely-benign from VUS (score = 0.2654, probability = 0.1) and VUS from likely-pathogenic (score = 0.7161, probability = 0.9). **c.** Frequency distribution of APOGEE 2 scores of Dataset 1's benign variants (training set) and Dataset 4's variants (ClinGen). Vertical dashed lines mark the average APOGEE 2 scores for both distributions.

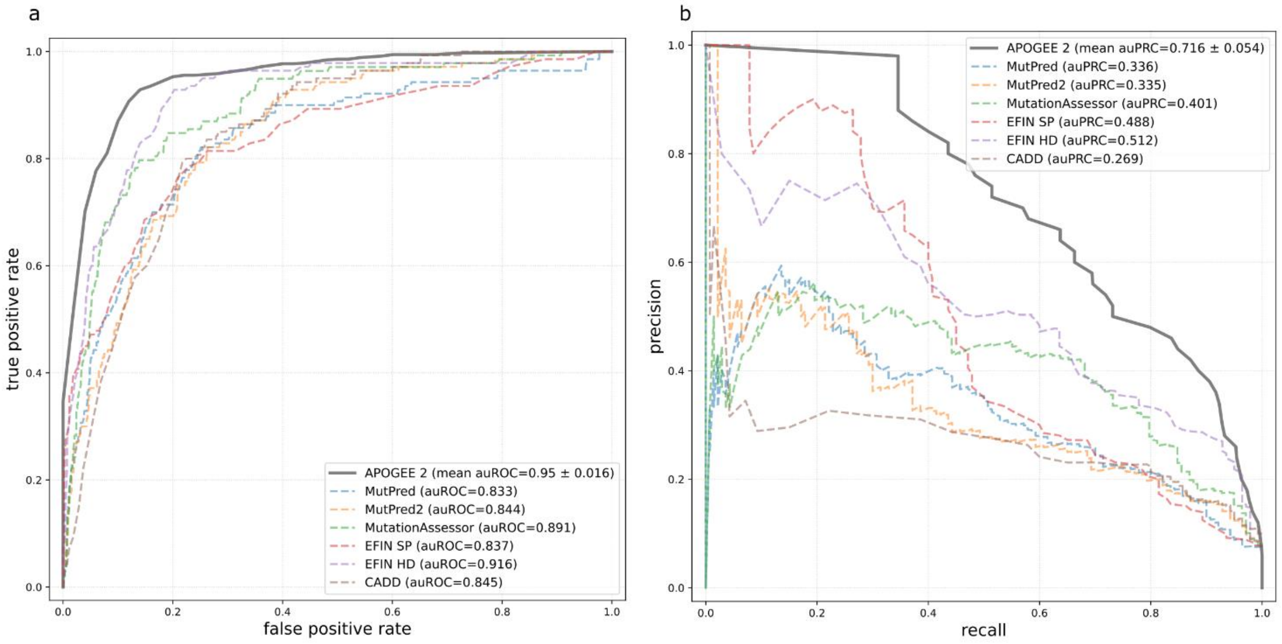

Supplementary Figure 3. Performance comparison of APOGEE 2 versus other predictors in terms of auROC (a) and auPRC (b). APOGEE 2's performance is reported as the mean ± 95% CIs obtained through cross-validation.

Supplementary Software 1

## Molecular Dynamics Simulation protocol
# Minimization and equilibration protocol generated by CHARMM-GUI (http://www.charmm-gui.org) v3.5
# GaMD production protocol implemented following https://miaolab.ku.edu/GaMD/manual.html guidelines
# All commands/inputs were implemented for AMBER20; a different version of AMBER may cause errors.

```
set amber = pmemd

set init = step5_input
set mini_prefix = step6.0_minimization
set equi_prefix = step6.%d_equilibration
set prod_prefix = step7_production
set prod_step   = step7
```

# Minimization

```
if (-e dihe.restraint) sed -e "s/FC/250.0/g" dihe.restraint > ${mini_prefix}.rest
pmemd -O -i ${mini_prefix}.mdin -p ${init}.parm7 -c ${init}.rst7 -o ${mini_prefix}.mdout
-r ${mini_prefix}.rst7 -inf ${mini_prefix}.mdinfo -ref ${init}.rst7
```

# Equilibration

```
set cnt = 1
set cntmax = 6
set fc = {'250.0','100.0','50.0','50.0','25.0'}

while ( ${cnt} <= ${cntmax} )
       @ pcnt = ${cnt} - 1
       set istep = `printf ${equi_prefix} ${cnt}`
       set pstep = `printf ${equi_prefix} ${pcnt}`
       if ( ${cnt} == 1 ) set pstep = ${mini_prefix}

       if (-e dihe.restraint && ${cnt} < ${cntmax}) sed -e "s/FC/${fc[${cnt}]}/g"
dihe.restraint > ${istep}.rest
       ${amber} -O -i ${istep}.mdin -p ${init}.parm7 -c ${pstep}.rst7 -o ${istep}.mdout -
r ${istep}.rst7 -inf ${istep}.mdinfo -ref ${init}.rst7 -x ${istep}.nc
       @ cnt += 1
end
```

# Gaussian accelerated Molecular Dynamics production

#Run initial conventional molecular dynamics and, after ntcmd+ntebsteps (defined in prep_gamd.in), run GaMD equilibration

```
pmemd.cuda -O -i prep_gamd.in -o 1_gamd_Prod.out -p step5_input.parm7 -c
step6.6_equilibration.rst7 -inf 1_gamd_Prod.info -x 1_gamd_Prod.mdcrd -r 1_gamd_Prod.rst
-gamd gamd_prod_prep.log
```

#start GaMD production simulation

```
pmemd.cuda -O -i 1_gamd.in -o 2_gamd_Prod.out -p step5_input.parm7 -c 1_gamd_Prod.rst -
inf 2_gamd_Prod.info -x 2_gamd_Prod.mdcrd -r 2_gamd_Prod.rst -gamd gamd_prod_1.log
```

#repeat running jobs using the same input file (${cnt}_gamd.in) until end of the GaMD production simulation

```
set cnt    = 3
set cntmax = 10
while ( ${cnt} <= ${cntmax} )
       pmemd.cuda -O -i  ${cnt}_gamd.in -o  ${cnt}_gamd_Prod.out -p step5_input.parm7 -
c  $((cnt-1))_gamd_Prod.rst -inf  ${cnt}_gamd_Prod.info -x  ${cnt}_gamd_Prod.mdcrd -
r  ${cnt}_gamd_Prod.rst -gamd gamd_prod_${cnt}.log
       @ cnt += 1
end
```

#Template input file (prep_gamd.in, 1_gamd.in, 2-10_gamd.in) for GaMD production steps

prep_gamd.in
```
&cntrl
imin = 0,
irest = 0,
ntx = 1,
ioutfm = 1,
nstlim = 50000000,
dt = 0.002,
ntt = 3,
gamma_ln = 5.0,
ig = -1,
tempi = 300.0,
temp0 = 300.0,
ntp = 0,
ntb = 1,
ntc = 2,
ntf = 2,
cut = 12,
ntwr = 500,
ntpr = 200,
ntwx = 5000,
ntwe = 200,
iwrap = 1,
ntr = 0,
ntwprt = 178474,

igamd = 3, iE = 1, irest_gamd = 0,
ntcmd = 10000000, nteb = 50000000, ntave =2000000,
ntcmdprep = 4000000, ntebprep = 4000000,
sigma0P = 12.0, sigma0D = 12.0,
&end
```

1_gamd.in
```
&cntrl
imin = 0,
irest = 0,
ntx = 1,
ioutfm = 1,
nstlim = 10000000,
dt = 0.002,
ntt = 3,
gamma_ln = 5.0,
ig = -1,
tempi = 300.0,
temp0 = 300.0,
ntp = 0,
ntb = 1,
ntc = 2,
ntf = 2,
cut = 12,
ntwr = 500,
ntpr = 200,
ntwx = 400,
ntwe = 200,
iwrap = 1,
ntr = 0,
ntwprt = 178474,

igamd = 3, iE = 1, irest_gamd = 1,
ntcmd = 0, nteb = 0, ntave = 2000000,
ntcmdprep = 0, ntebprep = 0,
sigma0P = 12.0, sigma0D = 12.0,
&end
```

2-10_gamd.in

```
&cntrl
imin = 0,
irest = 1,
ntx = 5,
ioutfm = 1,
nstlim = 10000000,
dt = 0.002,
ntt = 3,
gamma_ln = 5.0,
ig = -1,
tempi = 300.0,
temp0 = 300.0,
ntp = 0,
ntb = 1,
ntc = 2,
ntf = 2,
cut = 12,
ntwr = 500,
ntpr = 200,
ntwx = 400,
ntwe = 200,
iwrap = 1,
ntr = 0,
ntwprt = 178474,

igamd = 3, iE = 1, irest_gamd = 1,
ntcmd = 0, nteb = 0, ntave = 2000000,
ntcmdprep = 0, ntebprep = 0,
sigma0P = 12.0, sigma0D = 12.0,
&end
```
