# Supplementary material for: APOGEE 2: multi-layer machine-learning model for the interpretable prediction of mitochondrial missense variants
Source: Nat Commun. 2023 Aug 19;14:5058. doi: 10.1038/s41467-023-40797-7 (PMC10439926; doi:10.1038/s41467-023-40797-7)
Supplement: Supplementary file 3 — Description of Additional Supplementary Files [file 41467_2023_40797_MOESM3_ESM.pdf]

File Name: Supplementary Data 1

Description: GnomAD variants that are in MITOMAP as confirmed, those with an allele frequency greater than 0.002%, plus three gnomAD variants that were deemed pathogenic by ClinVar (accessed in October 2021). We also reported HelixMTdb variants showing an allele frequency ≥ 0.002% and a confirmed disease status according to MITOMAP.

File Name: Supplementary Data 2

Description: Correlation matrix of the evolution, pathogenicity predictions, and protein structural features used to train APOGEE 2. It includes three evolution measures, fourteen *in silico* pathogenicity predictors, and one protein structural feature. Pearson correlation coefficients are reported.

File Name: Supplementary Data 3

Description: Parameter grids tested through GridSearch CV for all the machine learning methods evaluated in this work. The best parameters found for the selected method are marked in bold.

File Name: Supplementary Data 4

Description: Performance metrics of all the machine learning methods evaluated in this work and performance comparison between APOGEE 2 and other meta-predictors. In the upper table, for each metric, we reported the average performance ± 95% confidence intervals, calculated on 100 test folds. In the lower table, we reported the average performance metrics for all the meta-predictors compared to APOGEE 2.

File Name: Supplementary Data 5

Description: List of all MitImpact variants annotated and categorized according to APOGEE 2. Variants belonging to the training set (Dataset 1) have been flagged as “P” or “N” referring to pathogenic or deemed benign variants, respectively.

File Name: Supplementary Data 6
Description: List of the most significantly high local spatial autocorrelated amino acids (adjusted LISA one-sided permutation p-value <0.01).

File Name: Supplementary Data 7
Description: Datasets 2, 3, and 4 containing benign or likely benign variants, used to evaluate the specificity of the APOGEE2 predictions. Datasets 2 and 3 were retrieved from gnomAD and HelixMTdb, respectively, considering missense variants not included in the training set, showing an allele frequency ≥0.002% (according to the source database). Dataset 4 was obtained from MITOMAP (https://mitomap.org/MITOMAP/Benign, accessed on September 7, 2022) and refers to non-synonymous curated variants not included in the training set and flagged as Benign or Likely-benign.

File Name: Supplementary Data 8
Description: List of possible synergistic variants in MITOMAP. We functionally annotated each variant and reported its classification according to APOGEE 2.

File Name: Supplementary Data 9
Description: Frequency of missing values of APOGEE 2 features in Mitimpact.

File Name: Supplementary Data 10

Description: auROC values calculated on 5 random partitions of the 2022 MITOMAP dataset, after training APOGEE 2 on seven MITOMAP sets retrieved from 2008 to 2020 every two years.
